# Short-Term Chromium-Stress-Induced Alterations in the Maize Leaf Proteome

**DOI:** 10.3390/ijms140611125

**Published:** 2013-05-27

**Authors:** Rong Wang, Fei Gao, Bing-Qian Guo, Ji-Chang Huang, Lei Wang, Yi-Jun Zhou

**Affiliations:** 1 College of Life Science, Fuyang Teachers College, Fuyang 236037, China; E-Mails: wangrbnu@yahoo.com.cn (R.W.); huangjichang1988@126.com (J.-C.H.); 2 College of Life and Environmental Sciences, Minzu University of China, Beijing 100081, China; E-Mails: gaofei@muc.edu.cn (F.G.); guobingqian2050@163.com (B.-Q.G.); 3 Biotechnology Research Institute, Chinese Academy of Agricultural Sciences, Beijing 100081, China

**Keywords:** maize, chromium, leaf, proteomics

## Abstract

Soil contamination by chromium (Cr) has become an increasing problem worldwide as a result of extensive industrial activities. Chromium, especially hexavalent Cr, impairs the growth and productivity of plants. Although it has been proposed that plants could modify their metabolism to adapt to Cr stress by reprogramming the expression of genes, especially those related to the antioxidant system, damage response, and electron transport chain, evidence at the protein expression level is lacking. To better understand the precise mechanisms underlying Cr phytoxicity and the plant response to Cr exposure, the time-course of changes in the protein expression profile induced by short-term hexavalent Cr exposure (1, 6 and 24 h) were analyzed in maize leaves. Among the over 1200 protein spots detected reproducibly by two-dimensional electrophoresis (2-DE), 60 were found to be differentially accumulated during Cr stress treatment. Of the Cr-regulated proteins, 58 were identified using tandem mass spectrometry (MS/MS). The Cr-regulated proteins identified were mainly involved in ROS detoxification and defense responses (26%), photosynthesis and chloroplast organization (22%), post-transcriptional processing of mRNA and rRNA (12%), protein synthesis and folding (10%), the DNA damage response (5%), and the cytoskeleton (3%). The possible involvement of these Cr stress-responsive proteins in Cr phytoxicity and the plant response to Cr exposure in maize is discussed, taking into consideration the information available from other plant models. Our results provide preliminary evidence that will facilitate understanding the molecular mechanisms underlying Cr toxicity in maize.

## 1. Introduction

Heavy metal contamination in soil is a cause of major environmental hazards globally, and results primarily from increased industrial pollution, urban activities, and agricultural practices [1]. Heavy metal pollution negatively affects the growth and development of plants, leading to losses of agricultural yields and endangering human health when it enters the food chain.

As one of the most abundant elements, chromium (Cr) exists in nature in both trivalent (Cr III) and hexavalent (Cr VI) forms, of which the latter is more toxic. Cr(VI) is highly hazardous to human health through inhalation, skin contact, and ingestion, being highly toxic, carcinogenic and mutagenic to living organisms, even when present in very low amounts. Excessive amounts of chromium within plants cause stunted growth of shoots and roots [2,3], lead to chlorosis of leaves, cell membrane damage, changes in the activity of various antioxidant enzymes and diminished photosynthesis [4]. Cr compounds released into the environment represent one of the most serious heavy metal pollutants both worldwide and in China [5]. Cr contamination originates mainly from industrial operations, including stainless steel production, mining, pigment manufacturing, petroleum refining, leather tanning, wood preservation, textile manufacturing, pulp processing, and fungicide development [5,6].

To cope with the detrimental effects of heavy metal accumulation, plants, like all other organisms, have evolved sophisticated mechanisms, including detoxification strategies based on chelation and subcellular compartmentalization [7]. First, plants exposed to elevated concentrations of heavy metal ions attempt to prevent or reduce uptake into root cells by restricting metal ions to the apoplast or by inhibiting root-to-shoot long-distance transport [8]; this mechanism occurs mainly in the root. Second, metal ions already in the cell are addressed using various storage and detoxification strategies, including metal chelation, transport, and sequestration into the vacuole. Finally, oxidative stress defense mechanisms are activated, with synthesis of stress-related proteins and signaling molecules.

Although the molecular and physiological mechanisms of plant responses to heavy metals, especially lead (Pb) and cadmium (Cd), have been focused upon in recent years, chromium (Cr, especially Cr(VI)) has attracted less attention from plant scientists [9], and the detoxification mechanism for chromium in plants is poorly understood [10]. The molecular mechanisms underlying plant responses to Cr stress and the defense-related signal transduction process have been investigated only partially. For example, a number of candidate genes potentially involved Cr tolerance were identified in four willow species using a cDNA-AFLP method [11]. The Cr (VI) stress response in roots has been analyzed at the transcriptomic and metabolomic levels in rice [12] and at the proteomic level in roots of *Miscanthus sinensis* [13].

Because of its economic importance, the wide range of its growing area, and the feasibility of combining extensive physiological, agronomic, and genetic studies, maize (*Zea mays* L.) was selected as the model plant for this study. Cr stress-induced phytotoxic lesions on maize plantlets have been described [14]. A previous proteomic study investigated proteome changes in chromium-treated maize plantlets, and identified 11 differently expressed proteins including some antioxidant enzymes, such as superoxide dismutase and 1-Cys peroxiredoxin [15]. However, as different tissues, such as leaf and root, respond differentially to environment stimuli [16], monitoring the molecular dynamics of maize leaf during Cr stress is probably one of the best approaches to deciphering the physiological mechanisms involved in the stress response.

To better understand the metabolic pathways implicated in the heavy metal stress response in maize, in the present study, the time-course of changes in soluble proteins elicited by short-term hexavalent chromium treatment in maize leaves were analyzed using a comparative proteomics approach. In total, 60 chromium stress-regulated proteins were determined and 58 of them were identified successfully by MS/MS analysis. The results not only identified a large number of differentially expressed proteins previously reported to be involved in the heavy metal stress response, but also revealed novel proteins that may play important roles in the Cr stress response.

## 2. Results

### 2.1. The Morphological and Physiological Responses in Maize Leaves Induced by Cr Treatment

**Figure 1:**
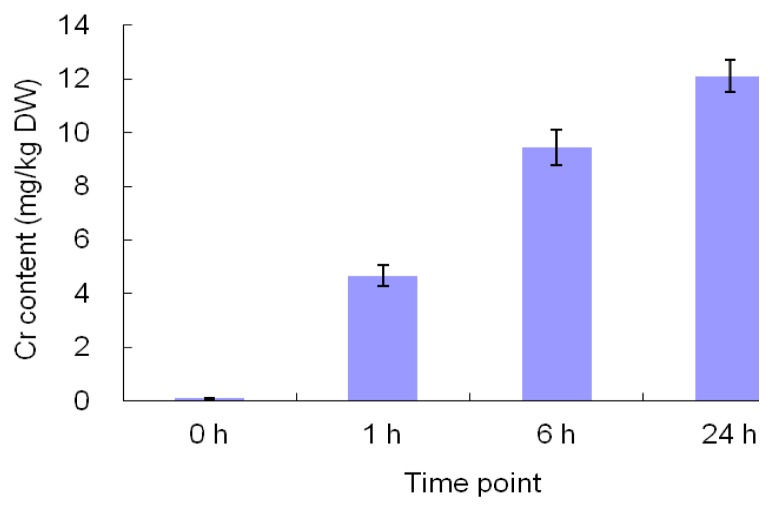
Effects of Cr treatment on Cr content of maize leaves. Maize seedlings were watered with 300 mg/L potassium dichromate solution for 24 h and the Cr concentrations of leaves were measured at the time point 0 h (prior to treatment), 1 h, 6 h and 24 h. The Cr levels are mean values ± SE.

Chromium accumulation in maize leaves increased with the duration of treatment (Figure 1). After 1 day of exposure to 300 mg/L potassium dichromate, the leaf tissue accumulated more than 12 mg/kg Cr on a dry weight basis, while that in the control plants was <0.2 mg/kg.

**Figure 2:**
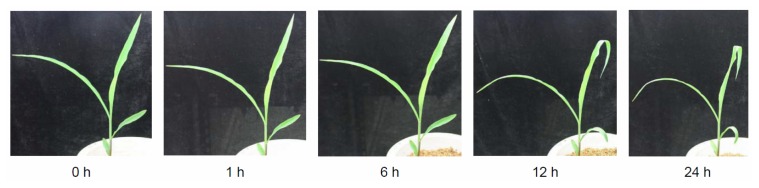
Effects of Cr stress on maize morphology. Maize seedlings were watered with 300 mg/L potassium dichromate for 24 h and photographed at 0, 1, 6, 12, and 24 h.

**Figure 3:**
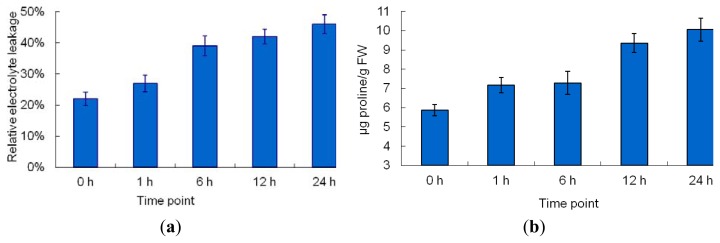
Physiological responses of maize leaves to Cr stress treatment. Maize seedlings were watered with 300 mg/L potassium dichromate for 24 h and the REL (**a**) and proline concentration (**b**) were measured at 0, 1, 6, 12, and 24 h.

The exposure of maize seedlings to short-term Cr stress resulted in obvious changes in morphology (Figure 2). The margins of young and middle leaves (the second, third, and fourth leaves) curled inward after 6 h of Cr treatment (Figure 2). Significant wilting of leaves was observed after 12 h of Cr treatment (Figure 2), possibly resulting from Cr treatment-induced water stress. Similar Cr stress-induced symptoms were reported previously [14]. Relative electrolyte leakage (REL) is an indicator of membrane damage caused by environmental stress. To estimate the effects of Cr stress-induced membrane damage in maize leaves, REL was measured in plants after exposure to Cr stress for 1, 6, 12, or 24 h. As shown in Figure 3a, the REL of maize leaves increased gradually, reaching a maximum at the 24-h time-point. Many types of environmental stress cause proline to accumulate to high levels in a number of plant species; thus, the proline content of Cr-stressed maize leaves was measured. The concentration of proline increased gradually with treatment duration, reaching a peak at the 24-h time-point (Figure 3b). Collectively, our results clearly demonstrated that the treatment regime used in this study increased the Cr concentration of maize leaves significantly, and caused marked damage to maize leaf cells.

#### 2.2. 2-DE Analysis of Cr-Treated Maize Leaf Proteins

**Figure 4:**
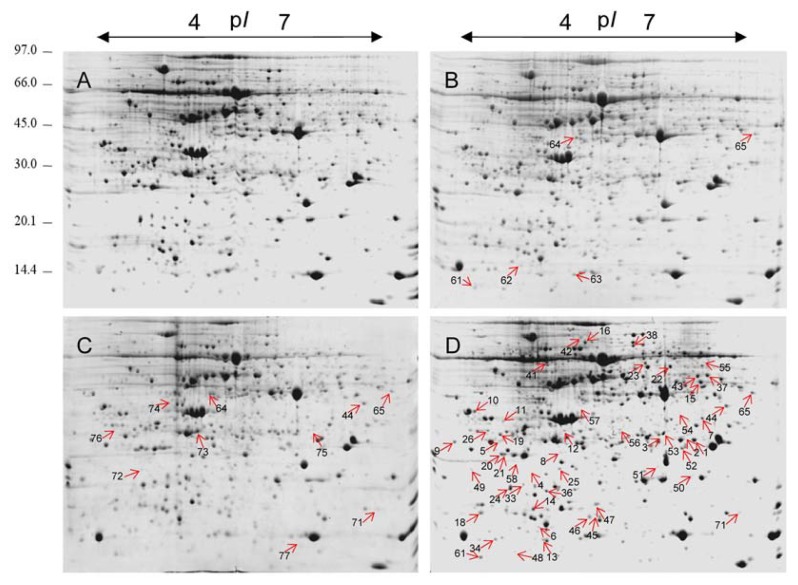
Representative images of 2-DE gels. Total leaf proteins were extracted and separated by 2-DE. Proteins (1200 μg) were separated in the first dimension on immobilized pH 4–7 pH dry strips (24 cm, linear) and in the second dimension on a 12.5% SDS-PAGE gel. The gel shown was stained with CBB-R250. Labeled spots indicate differentially expressed proteins showing at least a 1.5-fold change under Cr(VI) treatments, with *p* < 0.05. (**A**) 2-DE gel of control (0 h); (**B**) 2-DE gel of sample collected at the time-point 1 h; (**C**) 2-DE gel of sample collected at the time-point 6 h; (**D**) 2-DE gel of sample collected at the time-point 24 h.

The morphological and physiological data showed that the leaves of plants treated with potassium dichromate for 24 h showed stress symptoms. To identify the protein targets of Cr toxicity in maize leaves, total leaf proteins were extracted from control and Cr-treated leaves and separated by 2-DE. To investigate the dynamic protein expression patterns in response to short-term Cr(VI) stress, proteomic alterations in maize leaves after Cr(VI) treatment for 1, 6, and 24 h were examined. Representative 2-DE gels of the soluble protein fractions of leaf samples are shown in Figure 4. To gain a better resolution of total leaf proteins, 24-cm dry strips with a *pI* range of 4–7 were used. More than 1200 protein spots were reproducibly detected in each Coomassie brilliant blue (CBB)-stained gel using the ImageMaster Platinum 7.0 software. To evaluate the quantitative changes of each protein spot in the 2-DE gels, the relative spot volumes (% vol) were used. By software analysis, Student’s *t*-test (*p* < 0.05), coupled with a threshold of 1.5-fold change in abundance, revealed that 60 protein spots were differentially expressed in at least one time point during short-term potassium dichromate exposure (Figure S1).

**Figure 5:**
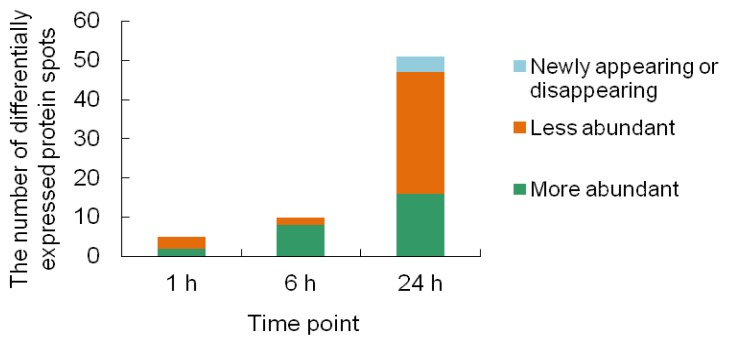
Distribution of protein spots upregulated, downregulated, and newly appearing or disappearing during Cr(VI) treatment.

Among the 60 differentially expressed protein spots, most showed quantitative changes; few spots (e.g., spots 1, 2, 50 and 54) either appeared or disappeared after Cr(VI) treatment. Of the Cr stress-responsive protein spots, five were identified at the 1-h time-point, 10 at the 6-h time-point, and 48 at the 24-h time-point (Figure 5). It is noteworthy that the majority of proteins (80%) were upregulated after Cr treatment for 6 h, while more proteins (61%) were downregulated after Cr treatment for 24 h.

### 2.3. Identification of the Differentially Expressed Proteins

Of the 60 differentially expressed protein spots, 58 (96.7%) were successfully identified using tandem mass spectrometry (MS/MS) and MASCOT searches, the abundance of two unidentified spots may have been too low for MS/MS analysis (Tables 1 and S1).

Some of the proteins identified were annotated as unknown and uncharacterized protein or as proteins without a specific function in the NCBI nr (green plants) database. To gain more information about functions and subcellular locations of these proteins, we searched them against known homologues in UniProtKB [17] and TAIR10 [18] with the BLASTP algorithm [19] using their amino acid sequences as queries. Homologues with the highest identity are shown in Table S1. All homologues in UniProtKB shared 100% positives with the corresponding proteins at the amino acid level, indicating that they are the same protein.

**Figure 6:**
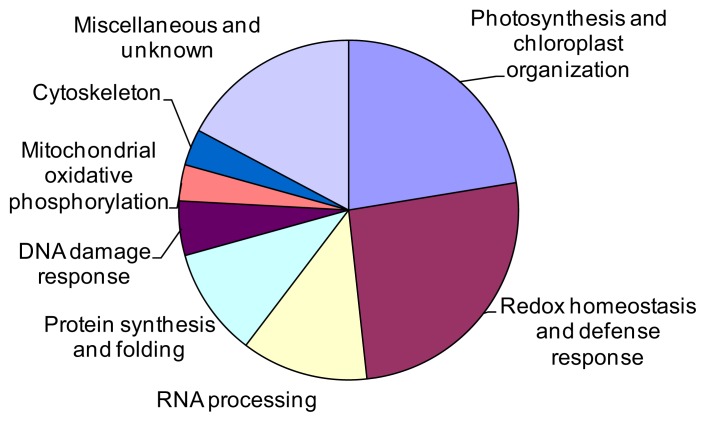
Functional distribution of 58 proteins differentially expressed in maize leaves under chromium stress. In total, eight functional groups are shown.

The 58 identified proteins spots were divided into eight groups according to their biological processes in UniProtKB [17] (Figure 6). The largest group was related to ROS detoxification and defense response (26%), including 15 Cr stress-responsive protein spots (spots 4, 8, 12, 20, 21, 22, 36, 37, 48, 51, 52, 53, 55, 56, 63, and 75, representing 14 unique proteins). The second-largest group contained 13 proteins (22%) associated with photosynthesis and chloroplast organization (spots 1, 2, 4, 7, 9, 16, 23, 26, 38, 44, 46, 50, and 73). The third group included seven proteins (12%) involved in post-transcriptional processing of mRNA and rRNA (spots 3, 5, 10, 11, 13, 15, and 19). The fourth group consisted of six proteins (10%) related to protein synthesis and folding (spots 6, 24, 41, 33, 47, and 61). The fifth group included three proteins (5%) associated with DNA damage response (spots 57, 64, and 71). The remaining three protein groups were involved in mitochondrial oxidative phosphorylation (spots 25 and 72), cytoskeleton (spots 34 and 45), and miscellaneous and unknown (spots 14, 18, 42, 49, 54, 58, 62, 65, 74, and 76).

Many Cr stress-responsive proteins identified in our experiments have already been discussed in the context of abiotic-stress responses. These include phosphomannomutase (spot 75) [20], 6-phosphogluconate dehydrogenase family protein (spot 55) [21], ATP sulfurylase (spot 22) [22], aspartate aminotransferase (spot 37) [23], peptide methionine sulfoxide reductase (spot 51) [24], 2-cys peroxiredoxin BAS1 (spots 20 and 21) [22], thioredoxin-dependent peroxidase 1 (TPX1) (spot 36) [25], thioredoxin X (spot 48) [25], cysteine proteinase inhibitor (spot 63) [22], glycine rich protein 2 (GRP2) (spots 3 and 13) [26], ubiquitin-conjugating enzyme E2 (UBC13B) (spot 71) [27], actin-depolymerizing factor 3 (ADF3) (spot 45) [28], filamentation temperature-sensitive H 2B (spot 16) [29], chloroplastic adenylate kinase (spot 73) [15], cytosolic ascorbate peroxidase (spot 53) [30], manganese superoxide dismutase (spot 52) [15], and chloroplastic thiamine thiazole synthase 1 and 2 (spots 57 and 64) [31]. Some proteins not previously reported to be involved in plant responses to abiotic-stress were identified, such as ribosome recycling factor (spots 1 and 2), glutamyl-tRNA(Gln) amidotransferase subunit C, chloroplastic/mitochondrial (spot 61), membrane steroid-binding protein 1 (spot 74), and profilin-5 (spot 34). Our results validate the efficacy of proteomics technologies or the identification and characterization of proteins involved in plant Cr stress responses.

## 3. Discussion

### 3.1. Photosynthesis and Chloroplast Organization

Photosynthesis is the most fundamental biological process in leaves. Previous studies revealed that chromium stress negatively affects chloroplast function, with toxic effects of: electron transport inhibition, reduced chlorophyll, calvin cycle enzyme inactivation, reduced CO_2_ fixation and chloroplast disorganization [9,14,32]. Not surprisingly, a large proportion (72%, 42/58) of the identified Cr-stress responsive proteins was predicted to be localized to the chloroplast in UniProtKB [17] (Table S1). These chloroplast-located Cr stress-responsive proteins are associated with various aspects of chloroplast structure and function, including proteins involved in photosynthetic electron transport, chloroplast organization and chlorophyll biosynthesis, and chloroplast redox homeostasis (thioredoxin X, 2-cys peroxiredoxin BAS1 and peptide methionine sulfoxide reductase), chloroplast RNA processing (ribonucleoprotein A), and chloroplast protein synthesis and folding (50S ribosomal protein L12-1 and CHL-CPN10). This result suggests that the regulation of chloroplast function is a central part of chromium stress responses in maize leaves.

It is well established Cr stress impairs electron transport [9]. In the present study, two subunits of ATP synthase (spot 9, ATP synthase delta chain; spot 46, ATP synthase epsilon chain, putative) were downregulated, possibly resulting in destabilization of the ATP synthase complex and a reduction of photophosphorylation.

The electron transport inhibition and chloroplast disorganization induced by Cr stress may result in photosynthetic apparatus damage, such as photosystem II (PSII) photodamage and chloroplast swelling. In this study, the up-regulation of a filamentation temperature-sensitive H 2B (spot 16) and a VIPP1 homologue (spot 7) under Cr stress may help to alleviate chloroplast structural damage. Filamentation temperature-sensitive H 2B (spot 16) is a metalloprotease that functions in thylakoid membrane biogenesis, and a recent report showed that the protein participates in repair of PSII following damaged incurred during photoinhibition [29]. The homologue of spot 7 in *Arabidopsis*, VIPP1 (a protein encoded by AT1G65260.1), is essential for thylakoid membrane formation, which was recently demonstrated to play a protective role in chloroplast envelope maintenance under stress condition [33].

Adenylate kinases equilibrate adenylates by the reversible formation of ADP through transfer of one phosphate group from ATP to AMP. The chloroplastic adenylate kinase is thought to play a vital role in the equilibration of adenylates and *de novo* synthesis of ADP in chloroplast, and the absence of AMK2 causes loss of chloroplast integrity [34]. The upregulation of chloroplastic adenylate kinase (spot 73) in this study may provide more adenylate substrate for ATP synthesis in stressed chloroplasts. Although upregulation of adenylate kinase induced by Cr stress of 72 h in maize plantlets had been reported previously [15], and adenylate kinase has been observed to be induced by drought stress in tomato [35], the relationship between adenylate kinase and environmental stress in plants remains unclear.

A ribosome recycling factor spot (spot 2), identified from 2-DE gels of control samples, was absent from 2-DE gels of 24-h Cr-treated samples, while a neighboring ribosome recycling factor spot (spot 1) with a similar molecular weight and a little higher isoelectric point emerged in gels of the 24-h Cr-treated samples, suggesting a possible post-translational modification of this protein induced by Cr stress. Because *Arabidopsis* chloroplast ribosome recycling factor was shown to play a critical role in chloroplast biogenesis [36], the changes in this protein observed here suggest that chloroplast ribosome recycling factor may be involved in chloroplast structure maintenance under Cr stress.

### 3.2. Defense Response and ROS Detoxification

Cr stress disturbs cellular redox homeostasis and promotes the production of reactive oxygen species (ROS), and plants activate ROS scavenging mechanisms to cope with oxidative stress [9]. Several proteins involved in cytosolic and chloroplast redox homeostasis were found to be regulated in the present study; this result is consistent with two previous Cr stress response studies [13,15].

Previous studies revealed that Cr stress promotes ROS accumulation partly by negatively affecting the electron transfer chains of the chloroplasts and mitochondria [37,38], yet the toxic effects at the molecular level remain incompletely understood. In the present study, several components of the electron transfer chains of the chloroplasts (discussed above) and mitochondria were found to be regulated by Cr stress, advancing our understanding of Cr toxicity to cellular redox homeostasis. Two proteins involved in the electron transfer chains of the mitochondria were identified: a mitochondrial ATP synthase D chain (spot 25), the abundance of which is downregulated, and a homologue of *Arabidopsis* NADH-ubiquinone oxidoreductase-related protein, which was upregulated by Cr stress. Additionally, apart from ROS generated by chloroplasts, mitochondria and peroxisomes, free radicals will be produced intracellularly during the reduction of Cr(VI) to Cr(III) [9]. All excess ROS must be eliminated by the cellular ROS detoxification systems.

Because of its inability to penetrate the membrane, O_2_^−^ produced in the mitochondrial electron transfer chains is converted to H_2_O_2_. The reaction is catalyzed by superoxide dismutases (SOD). SOD catalyzes the breakdown of superoxide radicals, O_2_^−^, and constitutes the first line of defense against ROS toxicity. In the present study, a mitochondrial manganese superoxide dismutase (SOD-3) was upregulated under Cr stress, suggesting an important role of SOD-3 in redox homeostasis control in maize leaf cells.

H_2_O_2_ is the most stable ROS molecule in cells, and the major sources of H_2_O_2_ include the electron transfer chains of the chloroplasts and mitochondria, as well as peroxisomes. Due to its ability to penetrate membranes freely, H_2_O_2_ is believed to be an ideal candidate for ROS signaling. Ascorbate peroxidase (APX) is the key H_2_O_2_ scavenging enzyme, other than catalase, which only catalyzes higher concentrations of H_2_O_2_. In our study, APX1 (spot 53) showed enhanced expression under Cr stress. Despite its cytoplasmic localization, APX1 has been suggested to play an important role in protecting chloroplasts against ROS [39].

The enhanced catalysis of H_2_O_2_ by APX1 may need higher levels of ascorbic acid (AsA). In the biosynthetic pathway for AsA in higher plants, phosphomannomutase (PMM) catalyzes the interconversion of mannose 6-phosphate and mannose 1-phosphate, and is involved in AsA biosynthesis and *N*-glycosylation [20,40]. Our results revealed that a homologue of *Arabidopsis* PMM (AT2G45790) showed elevated accumulation, indicating enhanced AsA synthesis in response to Cr stress.

In the ascorbate-glutathione pathway, glutathione (GSH) regenerates ascorbate by reducing dehydroascorbate (DHA). A protein (spot 22) that influences GSH level was upregulated in the present study, suggesting that more GSH is needed to diminish the elevated H_2_O_2_ via the ascorbate-glutathione pathway. Spot 22 is identified as bifunctional 3-phosphoadenosine 5-phosphosulfate synthetase 2 (ATP sulfurylase), which is the first enzyme in the sulfate assimilation pathway in plants. The enhanced expression of ATP sulfurylase under Cr stress may provide more GSH for ascorbate regeneration. More NADPH is required for the reduction of glutathione disulfide (GSSG) to GSH via glutathione reductase in plants under stress. A previous study suggested that the pentose phosphate pathway (PPP) plays an important role in plant responses to abiotic stresses; indeed, the second key enzyme of the PPP, 6-phosphogluconate dehydrogenase (6PGDH), may function as a regulator, controlling the efficiency of the PPP under abiotic stresses [21]. In the present study, we found a 6PGDH (spot 55) showing enhanced expression under Cr stress, indicating that PPP was enhanced under Cr stress. The enhanced PPP may provide more NADPH for GSH regeneration. We also found a 6-phosphogluconolactonase (PGL, spot 12), which was downregulated under Cr stress. Knockdown of PGL3, a 6-phosphogluconolactonase in *Arabidopsis*, leads to a significant increase in total glucose-6-phosphate dehydrogenase (G6PDH, the rate-limiting enzyme of the PPP) activity, resulting in a significantly higher total GSH level and the GSH/GSSG ratio [41]. Thus, downregulation of PGL in Cr-stressed maize leaves may enhance NADPH supply via promoting G6PDH activity.

Elevated ROS cause methionine (Met) oxidation to methionine sulfoxide (MetSO), which results in the modification of the activity and conformation of many proteins, and methionine sulfoxide reductase (MSR) catalyzes the reduction of MetSO back to Met. In the present study, a chloroplast MSR (spot 51) was upregulated by Cr stress, indicating involvement in the protection of chloroplasts against oxidative damage. Our result is consistent with a previous report that MSRs respond to drought, cold, and high light stress [24].

We also found two plant defense-related proteins (spot 63, cysteine protease inhibitor; spot 56, harpin binding protein 1) to be involved in Cr stress responses. Among them, cysteine protease inhibitors are involved in the regulation of protein turnover and play an important role in resistance against insects and pathogens [42], as well as tolerance to salt, drought, and cold stress [43]. Harpin binding protein induces plants to generate systemic acquired resistance (SAR) and has great biological significance in pest control [44].

### 3.3. Post-Transcriptional Processing of RNA

Recent advances in proteomics and metabolic profiling have suggested that, apart from the transcriptional regulation, post-transcriptional regulatory mechanisms also play key roles in plant abiotic stress responses [45]. RNA binding proteins bind RNA molecules immediately after transcription, forming mRNP complexes until completion of translation, participating in various steps of RNA processing and affecting the RNA population both quantitatively and qualitatively [46]. Our findings revealed that seven RNA binding proteins possibly involved in mRNA and rRNA processing were regulated by Cr stress, with six downregulated and one upregulated. Thus post-transcriptional processing of RNA in maize leaves may be impaired under elevated chromium conditions.

Among the various RNA-binding proteins, the glycine-rich RNA-binding proteins (GRPs) have been demonstrated to be involved in plant responses to a variety of environmental stresses, including heavy metals, cold, and drought stress [26]. A GRP1A and a GRP7 were found to be upregulated under cold stress [22], but downregulated in salt stress in *Thellungiella salsuginea* [47]; *Arabidopsis* GRP2, GRP4, and GRP7 affect seed germination, seedling growth, and stress tolerance of *Arabidopsis* plants under cold, salt, and dehydration stress conditions [48–50], and GRP7 has been shown to confer freezing tolerance by acting as a RNA chaperone in regulating export of mRNA [49]. In this study, two glycine-rich RNA-binding proteins (spots 3 and 13) were downregulated by Cr stress, indicating for the first time that Cr stress may negatively affect post-transcriptional processing of mRNA and rRNA. Additionally, Cr stress induces downregulation of GRP7 and ribonucleoprotein A, two proteins closely related to innate immune responses in plants [51], indicating that Cr stress may impair plant defense system.

### 3.4. Protein Synthesis and Folding

Heavy metal stress, or the oxidative stress induced by heavy metals, may affect protein synthesis apparatus, directly or indirectly [52]. Large quantities of misfolded proteins may accumulate in cells under stress conditions; higher levels of protein chaperones are needed to refold these proteins. In this study, six Cr-stress responsive protein spots, representing five proteins involving in protein synthesis and folding, were identified, including three ribosomal proteins (spots 24 and 33, chloroplast 50S ribosomal protein L12-1; spot 47, cytosolic 40S ribosomal protein S12), a protein involved in regulation of the translational fidelity (spot 61, Glu-tRNAGln amidotransferase, C subunit family protein), and two proteins participating in protein folding (spot 6, CHL-CPN10; spot 41, 40S ribosomal protein S16). Of the six protein spots, five were down-regulated and only one, spot 41, was upregulated. Our result is consistent with a previous study in which Cr stress induces upregulation of a proteins chaperone in *Miscanthus sinensis* [13]. These findings demonstrated involvement of regulation of protein translation and folding in the chromium stress response in maize leaves.

### 3.5. DNA Damage Response

Cr-induced DNA damage is thought to be caused directly by interactions between chromium and DNA [53], or indirectly, by Cr(VI)-induced oxidative stress (*i.e*., the elevated intracellular ROS concentration [54]), although the evidence is mainly from mammalian cells. In this study, three proteins that are likely related to DNA damage responses were regulated under Cr stress: putative ubiquitin-conjugating enzyme family (spot 71), thiamine thiazole synthase 2 (chloroplastic precursor) (spot 64), and thiamine thiazole synthase 1 (chloroplastic precursor) (spot 57), suggesting that the elevated Cr concentration induced DNA damage in maize leaves. A homologue of the putative ubiquitin-conjugating enzyme family (Zea mays) in *Arabidopsis thaliana* (identity = 98%), UBC36/UBC13B, encodes a protein that may play a role in DNA damage responses and error-free post-replicative DNA repair [27]. The homologues of the other two thiamine thiazole synthase chloroplastic precursors (*Zea mays*) in *Arabidopsis thaliana* (identities = 78% and 74%, respectively) may play important roles in adaptation to various stress conditions and in DNA damage tolerance [55]. This finding demonstrates for the first time the potential genotoxicity of Cr(VI) in plant cells.

### 3.6. Cytoskeleton

The actin cytoskeleton is critical for a variety of cellular processes. Two actin-cytoskeletonorganization- related proteins were downregulated under Cr stress: an actin-depolymerizing factor 3 (ADF3, spot 45) and a profilin-5 (spot 34). ADF3 has been reported to be responsive to various environmental stresses, such as salt, oxidative, and cadmium stress, in *Arabidopsis* [28,56]. Maize ADF3 localizes to a region where actin is being remodeled during tip growth [57], suggesting ADF3 may play a role in plant growth. Profilin binds to actin and affects the structure of the cytoskeleton in a concentration-dependent manner. At high concentrations, profilin prevents the polymerization of actin whereas at low concentrations, it enhances the polymerization. *Arabidopsis* PRF3 (formerly profilin-5, homologue of maize profilin-5) is strongly expressed in young seedlings and affects cell elongation and F-actin organization [58]. The downregulation of ADF3 and profilin-5 in maize leaves observed in this study is closely related to the growth inhibition induced by Cr stress reported previously [9].

### 3.7. Miscellaneous and Unknown Proteins

SOUL heme-binding protein is thought to function in heme transfer or heme binding to prevent damage by ROS [59]. A SOUL heme-binding protein (spot 54) and a heme-binding protein 2 (spot 76, a homologue of *Arabidopsis* SOUL heme-binding family) were found to be upregulated under Cr stress, suggesting their involvement in ROS detoxification. A membrane steroid-binding protein 1 (MSBP1, spot 74) showed decreased abundance under Cr stress. MSBP1 is believed to act as a negative factor at an early stage of brassinosteroids (BRs) synthesis [60]. Because BRs promote tolerance in plants to a wide range of stresses, including heat, cold, drought, and salinity [61], the downregulation of MSBP1 may lead to activation of the BR signaling pathway, leading to enhanced tolerance to Cr stress. Our results suggest for the first time that BR signaling may be involved in Cr stress response. Additionally, a mitochondrial ATP synthase D chain (spot 25) was downregulated and another component of the respiratory electron transport chain (spot 72) was upregulated, suggesting that Cr stress had a toxic effect on mitochondrial oxidative phosphorylation.

As a starting point to address the molecular mechanisms underlying the Cr stress induced physiological response in maize leaves, the present study identified Cr stress-responsive proteins, providing important data for understanding the Cr stress-induced response in maize leaves. However, a maize leaf consists of cells of various developmental stages, which exhibit differing metabolic activities, which may lead to accumulation of different amounts of Cr. Future studies should focus on the growth zone of the leaf, because cell production and expansion are restricted to that segment, to decipher the mechanism behind the Cr-induced leaf growth reduction. It is also noteworthy that the photosynthetic apparatus in C4 plants, including maize, is partitioned into two cell types, called the mesophyll (M) and bundle sheath (BS) cells, the differentiation of the specialized M and BS cells was recently achieved by a combination of microscopy and quantitative proteomics [62]. Investigation of the M- and BS-specific Cr-stress responses will provide deeper insight into C4-cell-specific adaptation mechanisms to environmental factors.

## 4. Experimental Section

### 4.1. Plant Growth and Chromium Treatment

Maize (*Zea mays* L.) seeds of the Zheng 58 inbred line were germinated in the dark at 25 °C on blotting paper saturated with deionized water. After 72 h, seedlings were transferred to 1-L pots filled with vermiculite, and placed in a growth chamber with a day/night regime of 16/8 h and a light intensity of 180 μmol m^−2^ s^−1^ at plant level, with a temperature of 22–24 °C in the dark and 25–27 °C in the light, with a relative humidity of 50%. Seedlings were watered every 4 days with half-strength Hoagland nutrient solution.

After the fourth leaves were fully developed, the plantlets were divided into four groups randomly, and three were watered with 300 mg/L potassium dichromate. The second and third leaves were excised from the seedlings after 1, 6 and 24 h, and the corresponding leaves separated from the untreated seedling were used as the control (0 h). All the leaf samples were snap-frozen in liquid nitrogen and stored at −80 °C.

### 4.2. Determination of Cr Accumulation

After treatment, leaf samples were washed with deionized water to remove surface Cr salts. The samples were dried in an incubator at 80 °C for 24 h, weighed, and then ground to a fine powder. Approximately 0.5 g of fine powder from each treatment group was digested, using a ternary solution (HNO_3_/H_2_SO_4_/HClO_4_, 10:1:4 *v*/*v*), and the total Cr in the digestion solution was determined with an atomic absorption spectrophotometer (Solaar M6, Thermo Fisher Scientific, Waltham, MA, USA). Three different biological replicate root samples were subjected to analysis.

### 4.3. Physiological Parameter Measurement

The relative electrolyte leakage (REL) assay was performed according to a method described previously [63]. Proline was determined according to Bates *et al*. [64]. Briefly, 0.5 g fresh weight of leaves was homogenized with 5 mL of 3% sulfosalicylic acid, and the homogenate was cooled after heating for 10 min at 100 °C. After centrifugation (4000× *g*, 10 min), the content of free proline in the supernatant was measured using Ninhydrin reagent at 520 nm and expressed as μg g^−1^ fresh weight. Eight replicates were performed for each sample.

### 4.4. Protein Extraction and Two-Dimensional Electrophoresis

The leaf proteins were extracted using a trichloroacetic acid/acetone method as described previously [22]. Protein concentration was determined using the Bradford assay (Bioteke, Beijing, China). For 2-DE, 1200 μg of extracted proteins were loaded onto semi-preparative gels. For isoelectric focusing, the Ettan IPGphor 3 isoelectric focusing system (GE Healthcare, Piscataway, NJ, USA) and pH 4–7 IPG strips (24 cm, linear) were used according to the manufacturer’s recommendations. The IPG strips were rehydrated for 13 h in 450 μL rehydration buffer containing protein samples. The gel strips were equilibrated for 15 min in 10 mL equilibration buffer (50 mM Tris-HCl buffer, pH 8.8, 6 M urea, 30% *v*/*v* glycerol, 2% *w*/*v* SDS, 1% *w*/*v* DTT and 0.002% *w*/*v* bromophenol blue). SDS-PAGE was performed with 12.5% gels using the Ettan Six system (GE Healthcare, Piscataway, NJ, USA). The gels were run at 5 W per gel for the first 30 min, followed by 17 W per gel.

Proteins in 2-DE gels were visualized by CBB R-250. The gels were scanned (400 dpi, 16-bit gray-scale pixel depth) using an UMAX 2100 scanner (Umax, Willich, Germany) in transmission mode as TIFF files and analyzed using the ImageMaster Platinum 7.0 software (GE Healthcare, Piscataway, NJ, USA). Each sample was analyzed by 2-DE in at least three repetitions for further analysis. The abundance of each protein spot was estimated by calculating the percentage volume (% vol). Only protein spots with significant and reproducible changes of at least 1.5-fold, and deemed significant by Student’s *t*-test at a level of 95%, were accepted as differentially expressed. These spots were then subjected to MS/MS analysis. The standard error (SE) was calculated from at least three spots in replicate gels.

### 4.5. In-Gel Digestion and Mass Spectrometry Analysis

Selected protein spots were excised manually from the CBB stained gels, and in-gel digestion was performed as reported previously [65]. Tryptic peptides were analyzed uaing an ABI 4800 MALDI-TOF/TOF Plus mass spectrometer (Applied Biosystems, Foster City, CA, USA). Data were acquired in a positive MS reflector using a CalMix5 standard to calibrate the instrument (ABI4800 Calibration Mixture). Both the MS and MS/MS data were integrated and processed using the GPS Explorer V3.6 software (Applied Biosystems, Foster City, CA, USA), with default parameters. Based on combined MS and MS/MS spectra, proteins were successfully identified based on 95% or higher confidence interval of their scores in the MASCOT V2.1 search engine (Matrix Science Ltd., London, UK), using the following search parameters: NCBI nr (green plants) database, trypsin as the digestion enzyme, one missed cleavage site, fixed modifications of carbamidomethyl (C), partial modification of acetyl (protein *N*-term), oxidation (M), deamidated:18O(1) (NQ), dioxidation (W), 120 ppm for precursor ion tolerance, and 0.3 Da for fragment ion tolerance.

## 5. Conclusions

In the present study, we investigated chromium stress induced physiological responses, and proteomic changes in maize leaves. A total of 60 proteins were identified that were differentially expressed in short-term chromium stress-treated leaf samples. The Cr stress-responsive proteins identified using MS analysis were mainly involved in ROS detoxification and defense responses, photosynthesis and chloroplast organization, post-transcriptional processing of mRNA and rRNA, protein synthesis and folding, DNA damage responses, and the cytoskeleton, suggesting that plants modify their metabolism by reprogramming the expression of genes to adapt to Cr stress. These findings might increase our understanding of the physiological response to Cr stress in maize leaves at the molecular level.

## Supplementary Information



## Figures and Tables

**Table 1:** Differentially expressed proteins under Cr stress conditions in maize leaves identified by MS analysis.

Spot ID	Accession number a	Protein name and plant species	Score	Theor. Mr/pI b	Exp. Mr/pI c
**Photosynthesis and chloroplast organization**					

23	gi|226508728	uncharacterized protein LOC100275158 [*Zea mays*]	414	51.9/6.12	53/5.9
44	gi|13096165	Chain A, Crystal Structure Of The Complex Between Ferredoxin And Ferredoxin-Nadp^+^ Reductase [*Zea mays*]	263	35.6/7.01	35/6.6
50	gi|194697374	unknown [*Zea mays*]	337	23.1/9.07	23/6.2
26	gi|226503027	uncharacterized protein LOC100272863 [*Zea mays*]	148	28.9/4.85	29/4.7
9	gi|195613650	ATP synthase delta chain [*Zea mays*]	335	26.7/4.73	27/4.2
46	gi|77554379	ATP synthase epsilon chain, putative [*Oryza sativa*]	151	15.3/5.46	16/5.4
38	gi|30575690	NADP-malic enzyme [*Zea mays*]	373	70.4/6.2	70/5.8
73	gi|1170606	Adenylate kinase, chloroplastic [*Zea mays*]	523	24.9/4.95	25/5.2
16	gi|187830110	filamentation temperature-sensitive H 2B [*Zea mays*]	335	72.6/5.69	73/5.4
7	gi|195622012	membrane-associated 30 kDa protein [*Zea mays*]	344	35.1/9.5	35/6.3
1	gi|226497262	ribosome recycling factor [*Zea mays*]	373	29.3/9.22	29/6.3
2	gi|226497262	ribosome recycling factor [*Zea mays*]	373	29.3/9.22	29/6.2
4	gi|226508836	uncharacterized protein LOC100277322 [*Zea mays*]	131	23.4/6.31	23/4.8

**Redox homeostasis and defense response**					

53	gi|226504576	APx1-Cytosolic Ascorbate Peroxidase [*Zea mays*]	764	27.5/5.65	27/5.9
52	gi|168624	manganese superoxide dismutase (SOD-3) [*Zea mays*]	407	25.6/7.11	25/6.1
75	gi|226530195	uncharacterized protein LOC100272867 [*Zea mays*	267	28.3/5.83	28/6.2
55	gi|194702230	unknown [*Zea mays*]	363	53.1/5.93	53/6.2
12	gi|414591366	6-phosphogluconolactonase isoform 1 [*Zea mays*]	371	34.8/7.71	31/5.2
21	gi|195626524	2-cys peroxiredoxin BAS1 [*Zea mays*]	81	28.3/5.81	28/4.5
20	gi|195626524	2-cys peroxiredoxin BAS1 [*Zea mays*]	89	28.3/5.81	28/4.5
36	gi|226505300	LOC100283392 [*Zea mays*]	165	17.3/4.85	18/4.9
8	gi|223943539	unknown [*Zea mays*]	309	28.1/8.79	28/5.2
48	gi|195624046	thioredoxin X [*Zea mays*]	213	19.3/8.75	14/4.8
63	gi|66866417	cysteine proteinase inhibitor [*Zea mays* subsp. parviglumis]	133	14.8/6.3	15/5.4
56	gi|293334301	uncharacterized protein LOC100383635 [*Zea mays*]	529	29.4/9.57	28/5.6
22	gi|226492878	bifunctional 3-phosphoadenosine 5-phosphosulfate synthetase 2 [*Zea mays*]	354	52.5/8.30	52/6.2
37	gi|226508814	aspartate aminotransferase [*Zea mays*]	306	50.5/8.15	50/6.4
51	gi|226532399	peptide methionine sulfoxide reductase [*Zea mays*]	775	20.8/5.85	21/5.9

**RNA processing**					

3	gi|363543235	uncharacterized protein LOC100857032 [*Zea mays*]	421	23.9/5.95	24/5.9
13	gi|195642478	glycine-rich RNA-binding protein 2 [*Zea mays*]	115	15.6/9.00	14/4.8
15	gi|414884012	hypothetical protein ZEAMMB73_274910 [*Zea mays*]	253	42.3/8.14	43/6.4
5	gi|226502782	ribonucleoprotein A [*Zea mays*]	123	28.5/4.83	29/4.6
19	gi|226502782	ribonucleoprotein A [*Zea mays*]	103	28.5/4.83	30/4.5
10	gi|162463757	nucleic acid binding protein1 [*Zea mays*]	274	33.2/4.6	33/4.4
11	gi|219363077	uncharacterized protein LOC100217196 [*Zea mays*]	284	31.5/5.13	31/4.6

**Protein synthesis and folding**					

1	gi|413915954	40S ribosomal protein S16, mRNA [*Zea mays*]	431	59.4/5.28	53/5.1
47	gi|195604208	40S ribosomal protein S12 [*Zea mays*]	180	15.4/5.33	18/5.4
61	gi|195647902	Glu-tRNAGln amidotransferase, C subunit family [*Zea mays*]	304	15.9/5.76	14/4.6
6	gi|414585580	CHL-CPN10 [*Zea mays*]	106	14.5/6.15	15/4.8
33	gi|195610950	50S ribosomal protein L12-1 [*Zea mays*]	147	19.1/5.4	19/4.9
24	gi|195659273	50S ribosomal protein L12-1 [*Zea mays*]	156	19.0/5.71	19/4.7

**DNA damage response**					

71	gi|414881042	putative ubiquitin-conjugating enzyme family [*Zea mays*]	377	17.3/6.74	17/6.7
64	gi|239985534	thiamine thiazole synthase 2, chloroplastic precursor [*Zea mays*]	432	37.4/5.59	38/5.3
57	gi|239985530	thiamine thiazole synthase 1, chloroplastic precursor [Zea mays]	279	37.3/4.87	34/5.3

**Cytoskeleton**					

34	gi|162461296	profilin-5 [*Zea mays*]	177	14.2/4.59	14/4.6
45	gi|162459533	actin-depolymerizing factor 3 [*Zea mays*]	490	16.0/5.46	16/5.4

**Mitochondrial oxidative phosphorylation**					

25	gi|226507194	ATP synthase D chain, mitochondrial [*Zea mays*]	178	19.9/5.19	23/5.2
72	gi|223973939	unknown [*Zea mays*]	246	24.3/5.68	24/4.8

**Miscellaneous and unknown**					

42	gi|414882068	putative alpha-L-arabinofuranosidase family protein [*Zea mays*]	303	73.0/5.1	73/5.2
76	gi|195638660	heme-binding protein 2 [*Zea mays*]	448	23.8/4.75	24/4.5
54	gi|226532343	SOUL heme-binding protein [*Zea mays*]	529	32.1/9.09	33/6.2
65	gi|226507242	uncharacterized protein LOC100274379 [*Zea mays*]	776	38.8/6.3	38/6.7
18	gi|226493727	uncharacterized protein LOC100275650 [*Zea mays*]	107	17.6/5.79	16/4.4
14	gi|302819846	Hypothetical protein SELMODRAFT_133757 [*Selaginella moellendorffii*]	131	17.2/5.22	16/4.8
49	gi|226508942	Uncharacterized protein LOC100275367 [*Zea mays*]	88	23.4/4.97	23/4.4
58	gi|226528599	Uncharacterized protein LOC100276423 [*Zea mays*]	215	19.8/4.71	23/4.9
74	gi|195635483	Membrane steroid-binding protein 1 [*Zea mays*]	425	27.9/5.45	28/5.0
62	gi|223948417	Unknown [*Zea mays*]	115	20.4/7.66	15/4.8

a Accession number in the NCBI nr (green plants) database;

b Theoretical molecular weight and isoelectric point;

c Experimental molecular weight and isoelectric point.
