# Supplementary material for: Short-Term Chromium-Stress-Induced Alterations in the Maize Leaf Proteome
Source: Int J Mol Sci. 2013 May 27;14(6):11125–44. doi: 10.3390/ijms140611125 (PMC3709723; doi:10.3390/ijms140611125)
Supplement: Supplementary file 1 [file ijms-14-11125-s001.pdf]

Supplementary Information

Figure S1. The expression profiles of the differentially accumulated protein spots during Cr stress treatment. The *X*-axis stands for time points. The *Y*-axis stands for the average relative spot volumes (%vol) of each protein spots and the data represent the mean values and SE of at least three independent experiments.

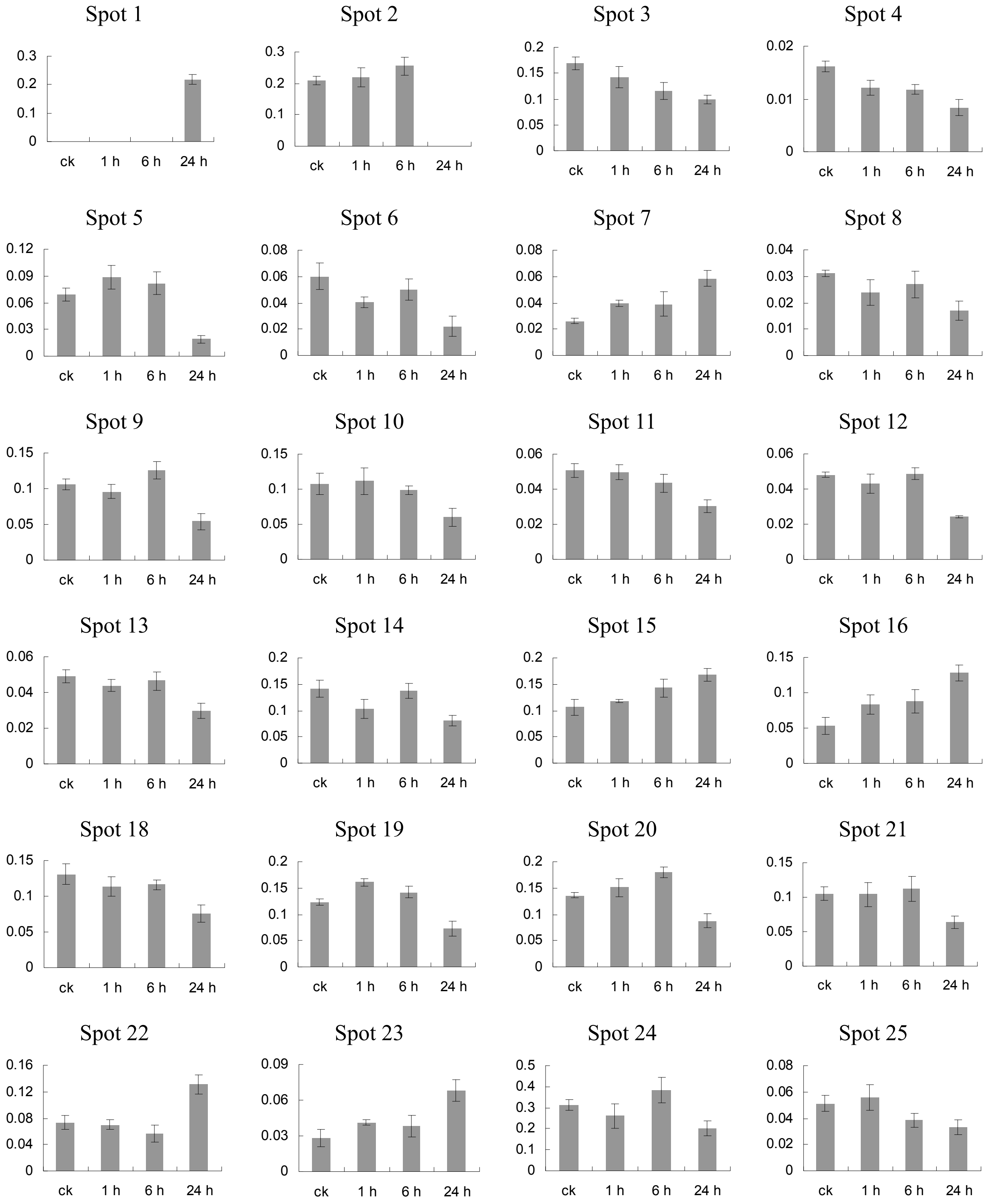

Figure S1. *Cont.*

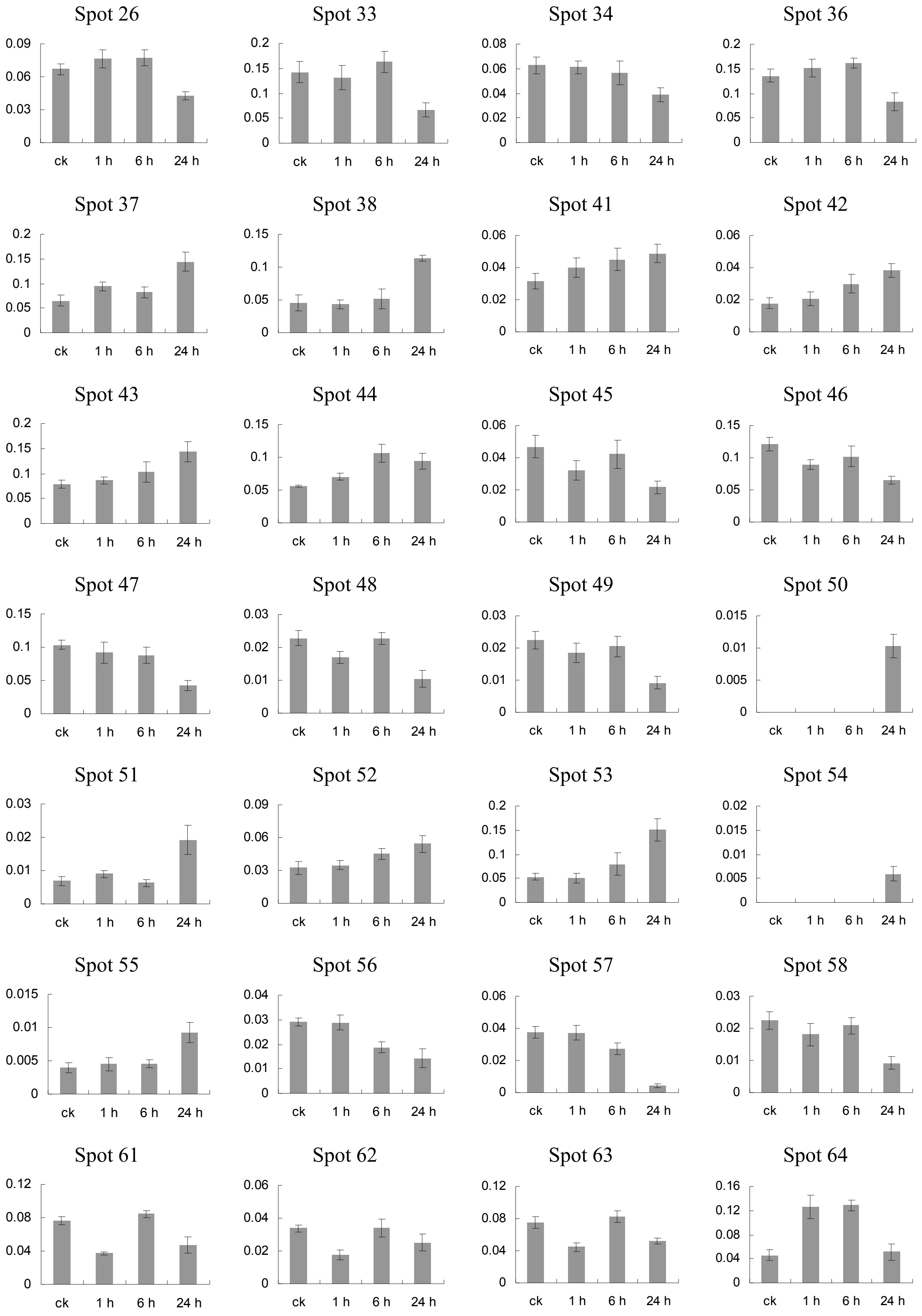

Figure S1. *Cont.*

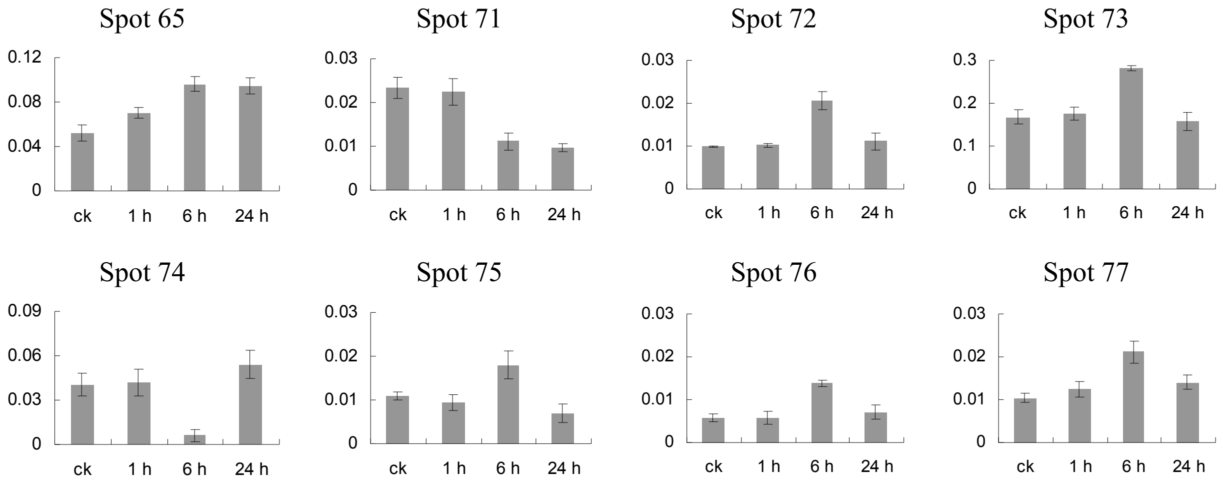

© 2013 by the authors; licensee MDPI, Basel, Switzerland. This article is an open access article distributed under the terms and conditions of the Creative Commons Attribution license (http://creativecommons.org/licenses/by/3.0/).
